# Impact of Extensively Hydrolyzed Infant Formula on Circulating Lipids During Early Life

**DOI:** 10.3389/fnut.2022.859627

**Published:** 2022-05-24

**Authors:** Santosh Lamichhane, Heli Siljander, Marja Salonen, Terhi Ruohtula, Suvi M. Virtanen, Jorma Ilonen, Tuulia Hyötyläinen, Mikael Knip, Matej Orešič

**Affiliations:** ^1^Turku Bioscience Centre, University of Turku and Åbo Akademi University, Turku, Finland; ^2^Pediatric Research Center, Children’s Hospital, University of Helsinki and Helsinki University Hospital, Helsinki, Finland; ^3^Research Program for Clinical and Molecular Metabolism, Faculty of Medicine, University of Helsinki, Helsinki, Finland; ^4^Health and Well-Being Promotion Unit, Finnish Institute for Health and Welfare, Helsinki, Finland; ^5^Faculty of Social Sciences, Unit of Health Sciences, Tampere University, Tampere, Finland; ^6^Center for Child Health Research and Research, Development and Innovation Centre, Tampere University Hospital, Tampere, Finland; ^7^Immunogenetics Laboratory, Institute of Biomedicine, University of Turku, Turku, Finland; ^8^School of Science and Technology, Örebro University, Örebro, Sweden; ^9^Department of Paediatrics, Tampere University Hospital, Tampere, Finland; ^10^School of Medical Sciences, Örebro University, Örebro, Sweden

**Keywords:** lipidome, early life, metabolomics, extensively hydrolyzed infant formula, intestinal permeability, lipidomics

## Abstract

**Background:**

Current evidence suggests that the composition of infant formula (IF) affects the gut microbiome, intestinal function, and immune responses during infancy. However, the impact of IF on circulating lipid profiles in infants is still poorly understood. The objectives of this study were to (1) investigate how extensively hydrolyzed IF impacts serum lipidome compared to conventional formula and (2) to associate changes in circulatory lipids with gastrointestinal biomarkers including intestinal permeability.

**Methods:**

In a randomized, double-blind controlled nutritional intervention study (*n* = 73), we applied mass spectrometry-based lipidomics to analyze serum lipids in infants who were fed extensively hydrolyzed formula (HF) or conventional, regular formula (RF). Serum samples were collected at 3, 9, and 12 months of age. Child’s growth (weight and length) and intestinal functional markers, including lactulose mannitol (LM) ratio, fecal calprotectin, and fecal beta-defensin, were also measured at given time points. At 3 months of age, stool samples were analyzed by shotgun metagenomics.

**Results:**

Concentrations of sphingomyelins were higher in the HF group as compared to the RF group. Triacylglycerols (TGs) containing saturated and monounsaturated fatty acyl chains were found in higher levels in the HF group at 3 months, but downregulated at 9 and 12 months of age. LM ratio was lower in the HF group at 9 months of age. In the RF group, the LM ratio was positively associated with ether-linked lipids. Such an association was, however, not observed in the HF group.

**Conclusion:**

Our study suggests that HF intervention changes the circulating lipidome, including those lipids previously found to be associated with progression to islet autoimmunity or overt T1D.

**Clinical Trial Registration:**

[Clinicaltrials.gov], identifier [NCT01735123].

## Introduction

Breast milk is an optimal food for early nutrition of infants. Exclusive breastfeeding is recommended during the first 6 months of life by the World Health Organization (WHO); however, 60–70% of infants worldwide are not exclusively breastfed for the recommended period (1, 2). Infant formulas (IF) are the common alternative to breast milk. IFs are typically prepared from industrially modified cow’s milk and processed to adjust its nutritional content according to the needs of infants (3). Several modifications to regular IF, such as adding prebiotics, probiotics, or modulating protein content, are performed to meet the specific nutritional demands of infants (4, 5). The protein content can be modified by hydrolyzation, where the proteins are hydrolyzed to smaller peptides than those found in standard cow’s milk formula (3). Extensively hydrolyzed formula is a type of cow milk-based formula, which contains substantially higher concentrations, and the most diverse profile, of free amino acids (6). Despite the obvious modification, our understanding of how IF composition affects infant metabolism in the short- and long-term remains limited (4).

Cow’s milk protein allergy is one of the most common food allergies in infants and young children (7). Growing evidence suggests that early-life exposures to complex proteins, including cow’s milk proteins, may increase the risk of islet autoimmunity, particularly in genetically susceptible children (8–10). Meanwhile, weaning to IF with hydrolyzed milk proteins is hypothesized to protect the children from developing allergies and islet autoimmunity (11, 12). One possible protective mechanism may be improved *via* intestinal barrier function (13), which may subsequently prevent absorption of harmful, ingested substances, particularly pro-inflammatory molecules, microorganisms, toxins, and antigens (14). However, there are also reports that do not show any benefit of hydrolyzed IF as compared to conventional IF (15, 16).

Dietary intake influences metabolic profiles in children. Previous metabolomics-based studies showed that the type of feeding, e.g., breastfeeding or formula feeding, had a profound impact on infant blood lipid levels (5, 17, 18). Infant metabolic profiles have also been associated with multiple, health-related endpoints, e.g., gastrointestinal development, growth, neurological or cognitive development, and maturation of the immune system (19). Previous studies also suggest that lipid metabolism might be compromised in the period preceding the appearance of islet autoimmunity (20–22). Current knowledge about the impact of extensively hydrolyzed casein formula on the infant’s serum metabolome is limited. A metabolomics-based approach can be utilized to study diet-related differences (i.e., an extensively hydrolyzed formula vs. a conventional formula) and health outcomes.

Here, we hypothesized that extensively hydrolyzed milk formula alters intestinal permeability and pro-inflammatory metabolites, as compared to a conventional cow’s milk formula. Our aim was to compare longitudinal lipid profiles of infants fed extensively hydrolyzed infant formula (HF) vs. the conventional, regular infant formula (RF), examining these lipid profiles as regards intestinal function and growth of the infants.

## Materials and Methods

### Study Population

Pregnant Finnish women were recruited from 28 January 2013 to 26 February 2015, in the context of the Early Dietary Intervention and Later Signs of Beta-Cell Autoimmunity: Potential Mechanisms (EDIA) study, which is a small-scale randomized double-blind infant feeding trial comparing weaning infants onto an extensively hydrolyzed milk formula vs. a conventional, cow’s milk-based formula. Families were contacted at the time of the fetal ultrasonography visit, which is arranged for all pregnant women in Finland around gestational week 20. Written informed consent was signed by the parents to permit analysis of their human leucocyte (HLA) genotype to exclude infants without HLA-conferred susceptibility to T1D. At this point, 68% of the infants to be born were excluded. Separate informed consent was obtained from eligible parents at the beginning of the third trimester to analyze the offspring’s genotype and to continue in the intervention study. The cord blood from 309 newborn infants was screened to determine their HLA genotype, as previously described (23). Eligible genotypes, determined as previously described (24), were the high-risk genotype combining the (DR3)-DQA1* 05-DQB1*02 and DRB1*04:01/2/4/5-DQA1*03-DQB1*03:02 and the moderate-risk genotypes defined as homozygosity for either of the above, DRB1*04:01/2/5-DQA1*03-DQB1*03:02 with some of the selected neutral haplotypes, or the (DR3)-DQA1*05-DQB1*02/(DR9)-DQA1*03-DQB1*03:03 genotype. Selected neutral haplotypes included (DR1/10)-DQB1*05:01, (DR8)-DQB1*04, (DR7)-DQA1*02:01-DQB1*02, (DR9)-DQA1*03-DQB1*03:03, and (DR13)-DQB1*06:04. A total of eighty-seven offspring were eligible and 73 joined the intervention trial. Serum samples were collected at 3, 9, and 12 months of age. Child growth (weight and length) and intestinal function, including lactulose mannitol (LM) ratio, fecal calprotectin, and fecal β-defensin levels, were also measured at given time points. The study design was recently reported (25). Selected characteristics of the study subjects are shown in Table 1.

**TABLE 1:** Demographic characteristics of the study population.

Parameter	Median (range)
Maternal delivery age (years)	32.6 (20.9–45.8)
Gestational age (weeks)	39.7 (36.4–42.3)
Sex of the baby (male/female)	35/38
**Child weight (kg)**	
0 months (birth)	3.5 (4.72–2.6)
3 months	6.2 (8.2–4.3)
6 months	8.0 (5.8–10.6)
9 months	8.9 (7.1–11.7)
12 months	9.7 (7.3–13.2)
**Child length (cm)**	
0 months (birth)	50.1 (46.0–55.0)
3 months	62.2 (51.5–67.5)
6 months	68.0 (63.0–75.0)
9 months	72.7 (68.2–77.2)
12 months	76.4 (68.7–81.7)

### Analysis of Molecular Lipids

#### Sample Extraction

About 10 μl of serum was extracted with a modified version of the previously published Folch procedure (26). In short, 10 μl of 0.9% NaCl and 120 μl of CHCl3: MeOH (2:1, v/v) containing the internal standards (c = 2.5 μg/ml) were added to the sample.

The internal standard solution contained the following compounds: 1,2-diheptadecanoyl-sn-glycero-3-phosphoethanolamine [PE(17:0/17:0)], *N*-heptadecanoyl-D-erythro-sphingosylphosphorylcholine [SM(d18:1/17:0)], *N*-heptadecanoyl-D-erythro-sphingosine [Cer(d18:1/17:0)], 1,2-diheptadecanoyl-sn-glycero-3-phosphocholine [PC(17:0/17:0)], 1-heptadecanoyl-2-hydroxy-sn-glycero-3-phosphocholine [LPC(17:0)], and 1-palmitoyl-d31-2-oleoyl-sn-glycero-3-phosphocholine [PC(16:0/d31/18:1)], were purchased from Avanti Polar Lipids, Inc. (Alabaster, AL, United States), and triheptadecanoylglycerol [TG(17:0/17:0/17:0)] and cholest-5-en-3b-yl (heptadecanoate) [CE(17:0)] were purchased from Larodan AB (Solna, Sweden). The samples were vortex-mixed and incubated on ice for 30 min after which they were centrifuged (9,400 × *g*, 3 min). About 60 μl from the lower layer of each sample was then transferred to a glass vial with an insert, and 60 μl of CHCl3: MeOH (2:1, v/v) was added to each sample. The samples were stored at –80°C until analysis.

Calibration curves using 1-hexadecyl-2-(9Z-octadecenoyl)-sn-glycero-3-phosphocholine {PC[16:0e/18:1(9Z)]}, 1- (1Z-oc- tadecenyl)-2-(9Z-octadecenoyl)-sn-glycero-3-phosphocholine {PC[18:0p/18:1(9Z)]}, 1-stearoyl-2-hydroxy-sn-glycero-3- phos-phocholine [LPC(18:0)], 1-oleoyl-2-hydroxy-sn-glycero-3-phosphocholine [LPC(18:1)], 1-palmitoyl-2-oleoyl-sn-glycero-3-phosphoethanolamine [PE(16:0/18:1)], 1-(1Z-octadecenyl)-2-docosahexaenoyl-sn-glycero-3-phosphocholine [PC(18:0p/22:6)], and 1-stearoyl-2-linoleoyl-sn-glycerol [DG(18:0/18:2)], 1-(9Z-octadecenoyl)-sn-glycero-3-phospho-ethanolamine [LPE(18:1)], *N*-(9Z-octadecenoyl)-sphinganine {Cer[d18:0/18:1(9Z)]}, 1-hexadecyl-2-(9Z-octadecenoyl)-sn- glycero-3-phosphoethanolamine [PE(16:0/18:1)] from Avanti Polar Lipids, 1-palmitoyl-2-hydroxy-sn-glycero-3-phos-phatidylcholine [LPC(16:0)], 1,2,3 trihexadecanoalglycerol [TG(16:0/16:0/16:0)], 1,2,3-trioctadecanoylglycerol [TG(18:0/18:0/18:)] and 3β-hydroxy-5-cholestene-3-stearate [ChoE(18:0)], 3β-Hydroxy-5-cholestene-3-linoleate [ChoE(18:2)] from Larodan, were prepared to the following concentration levels: 100, 500, 1,000, 1,500, 2,000, and 2,500 ng/ml (in CHCl3:MeOH, 2:1, v/v) including 1,250 ng/ml of each internal standard.

#### Instrumental Analysis

The lipidomic analyses were performed using an ultra-high-performance liquid chromatography quadrupole time-of-flight mass spectrometry method (UHPLC-Q-TOF-MS from Agilent Technologies) (Santa Clara, CA, United States). The analysis was carried out on an ACQUITY UPLC BEH C18 column (2.1 mm × 100 mm, particle size 1.7 μm) by Waters (Milford, United States). An internal standard mixture was used for normalization, and lipid class-specific calibration was used for quantitation as previously described (24). Mass spectrometry (MS) data processing was performed using open source software MZmine 2.53 (27).

#### Data Pre-processing

Peak detection with a noise level of 1,000 was performed first, following with automated data analysis pipeline (ADAP) chromatogram builder including group intensity threshold to be as noise level (1,000), minimum highest intensity 10, and m/z tolerance 0.009 m/z or 8 ppm. Next, chromatogram deconvolution was performed with local minimum search as algorithm with a 70% chromatographic threshold, 0.05 min of minimum retention time range, 5% minimum relative height, 1,500 minimum absolute height, 1.05 as minimum ratio of peak top/edge, and peak duration range from 0.05 to 0.5 min. Isotopic peak grouper was done next with m/z tolerance of 0.06 m/z or 6 ppm, tR tolerance was set on 0.06 min, and a maximum charge of 2. For the alignment of peak lists, a Join alignment algorithm was performed with m/z tolerance as 0.008 or 10.0 ppm and a weight of m/z as 2 with a tR tolerance of 0.15 and a weight of tR 1. Next, filtering with feature list rows filter was done with three steps. First step rows that match with all criteria were kept with a retention time range from 2 to 12 and m/z of 369–1,200. Second filtering step removed rows that match with all criteria with a tR range of 2–4 and m/z of 800–1,200. Third filtering step removed rows that match with criteria with a tR range of 4–8 and m/z of 370–500. Next, gap-filling with peak finder was done with m/z tolerance of 0.006 m/z or 10.0 ppm, tR tolerance of 0.1 min, and intensity tolerance as 50%. Finally, the identification with a custom database was done to identify the peak list with compound names.

#### Quantitation and Quality Control

Quantification of lipids was performed using a 7-point internal calibration curve ranging from 0.1 up to 5 μg/ml. Quality control was performed throughout the dataset by including blanks, pure standard samples, extracted standard samples, and control plasma samples. Relative standard deviations (%RSDs) for lipids in the pooled samples (*n* = 7) were on average 11.8%.

#### Bile Acid Analyses

About 40 μl of serum sample was mixed with 90 μl of cold MeOH/H2O (1:1, v/v) containing the internal standard mixture (valine-d8, glutamic acid-d5, succinic acid-d4, heptadecanoic acid, lactic acid-d3, citric acid-d4, 3-hydroxybutyric acid-d4, arginine-d7, tryptophan-d5, glutamine-d5, 1-D4-CA,1-D4-CDCA,1-D4-CDCA,1-D4-GCA,1-D4-GCDCA,1-D4-GLCA,1-D4-GUDCA,1-D4-LCA,1-D4-TCA, and 1-D4-UDCA) for protein precipitation. The tube was vortexed and ultrasonicated for 3 min, followed by the centrifugation (10,000 rpm, 5 min). After centrifuging, 90 μl of the upper layer of the solution was transferred to the liquid chromatography (LC) vial and evaporated under the nitrogen gas to the dryness. After drying, the sample was reconstituted into 60 μl of MeOH: H2O (70:30).

Analyses were performed on an Acquity UPLC System coupled to a triple quadrupole mass spectrometer (Waters Corporation, Milford, United States) with an atmospheric electrospray interface operating in negative ion mode. Aliquots of 10 μl of samples were injected into the Acquity UPLC BEH C18 2.1 mm × 100 mm, 1.7-μm column (Waters Corporation). The mobile phases consisted of (A) 2 mM NH4Ac in H2O: MeOH (7:3) and (B) 2 mM NH4Ac in MeOH. The gradient was programmed as follows: 0–1 min, 1% solvent B; 1–13 min, 100% solvent B; 13–16 min, 100% solvent B; 16–17 min, 1% solvent B, flow rate 0.3 ml/min. The total run, including the reconditioning of the analytical column, was 20 min.

Quantification of bile acids (BAs) and per- and polyfluoroalkyl substances (PFAS) was performed using a 7-point internal calibration curve. The identification was done with a custom database, with identification levels 1 and 2, based on Metabolomics Standards Initiative.

### Gut Microbiome Analysis by Shotgun Metagenomics

These methods were adapted versions of descriptions in the related work (28).

#### Sample Collection and DNA Extraction

The stool samples were collected at home or in the delivery hospital. The samples collected at home were stored in the households’ freezers (−20°C) until the next visit to the study center. The samples were then shipped on dry ice to the EDIA Core Laboratory in Helsinki, where the samples were stored at −80°C until shipping to the University of Tampere for DNA extraction. DNA extractions from stool were carried out using the vacuum protocol of PowerSoil DNA Isolation Kit.

#### Metagenome Library Construction

Metagenomic DNA samples were quantified by Quant-iT PicoGreen dsDNA Assay (Life Technologies) and normalized to a concentration of 50 pg μl-1. Illumina sequencing libraries were prepared from 100–250 pg DNA using the Nextera XT DNA Library Preparation kit (Illumina) according to the manufacturer’s recommended protocol, with reaction volumes scaled accordingly. Batches of 24, 48, or 96 libraries were pooled by transferring equal volumes of each library using a Labcyte Echo 550 liquid handler. Insert sizes and concentrations for each pooled library were determined using an Agilent Bioanalyzer DNA 1000 kit (Agilent Technologies).

### Whole-Genome Sequencing

Whole-genome sequencing (WGS) libraries were sequenced on the Illumina HiSeq 2500 platform, targeting ∼2.5 Gb of sequence per sample with 101-bp paired-end reads (number of reads per sample is mentioned in Supplementary Table 1). Reads were quality-controlled by trimming low-quality bases and removing reads shorter than 60 nucleotides. Potential human contamination was identified and filtered using the KneadData Tool, v0.5.1 with the hg19 human reference genome. Quality-controlled samples were profiled taxonomically using MetaPhlAn 2.0 (29).

### The Lactulose–Mannitol Test

The infants were given an oral dose of 2 ml/kg of a lactulose–mannitol solution containing 5 g lactulose and 2 g mannitol per 100 ml after fasting for a minimum of 4 h. Urine was collected with specialized urine collection bags for 5 h. After measuring the total collected urine volume, the sample was stored in plastic tubes at –20°C.

Lactulose concentrations were measured as described by Northrop et al. (30) with the following modifications: sample and enzyme volumes 25 μl of sample or standard, 12.5 μl of β-galactosidase, 680 μl enzyme cocktail, and 20 μl of pepsinogen I (PGI). The final concentrations in the solutions were 500 U/ml for β-galactosidase, 10 mM for ATP, 14.9 mM for NADP, 3.64 U/ml for HK/G6P-DH, and 350 U/ml for PGI. Enzyme reactions were performed in plastic tubes and absorbance measurements in 96-well plates. A VICTOR, Wallac 1420 workstation (PerkinElmer, Waltham, MA, United States) was used for measuring the absorbances.

Mannitol concentrations were determined as described by Blood et al. (31) with the following modifications: sample and enzyme volumes 5 μl of sample or standard, 250 μl of enzyme cocktail, and 12.5 μl of mannitol dehydrogenase. The final concentrations in the solutions were 6.25 mM for NAD+, 6.55 mM for ATP, 3.64 U/ml for HK/G6P-DH, and 133 U/ml for mannitol dehydrogenase. Samples were analyzed in 96-well plates. The provider for mannitol dehydrogenase was Megazyme (Wicklow, Ireland), whereas all other reagents were purchased from Sigma-Aldrich (Darmstadt, Germany). After calculating the proportions of the excreted lactulose and mannitol, LM ratio was calculated by dividing the lactulose value by the mannitol value.

### Analysis of Fecal Calprotectin and Fecal Beta-Defensin

Fecal calprotectin and beta-defensin levels were analyzed from stool samples with commercial ELISA kits according to the manufacturer’s instructions (Calpro AS, Lysaker, Norway, and β-Defensin 2 ELISA Kit, Immundiagnostik, Bensheim, Germany) (32, 33). Briefly, approximately 100 mg of feces was obtained from each frozen sample. Extraction buffer was then added at a dilution of 1:50 for both HBD-2 and calprotectin. Fecal material with the extraction buffer was vortexed for 30 s, and mixing was continued in a shaker at 1,000 rpm for 3 min or until solid particles had dissolved. Samples were then centrifuged for 10 min at 10,000 *g* at room temperature, and the supernatants were collected and stored at −20°C until measured.

### Statistical Analysis

The lipidomic dataset was analyzed both at the individual lipid levels (each lipid as a single feature) and at the lipid class level [e.g., triacylglycerols (TGs)]. Lipid data values were log-transformed prior to multivariate analysis. For class-based analysis, individual lipid concentrations within each lipid class were added, and then, subsequent data analysis considering each lipid class as a variable was performed. The difference in the lipidome between the two intervention groups was compared using a multivariate linear model with case (HF vs. RF), co-accounting for sex (male vs. female) and amount of study formula per day (AFPD) using MaAsLin2 package in R (lipids ∼ sex + case + AFPD). Only those children with information about the AFPD were included in the multivariate linear comparative analysis. AFPD was calculated using the estimated amount of formula used during intervention (in grams) divided by the duration of regular use of study formula (in days). Adjusted *p*-values with FDR = 0.25 were considered significant. Spearman correlation coefficients were calculated using the Statistical Toolbox in MATLAB 2017b and *p*-values < 0.05 (two-tailed) were considered significant for the correlations. The individual Spearman correlation coefficients (R) were illustrated as a heat map using the ‘‘corrplot’’ package (version 0.84) for the R statistical programming language^1^. To subsequently visualize lactulose mannitol level, violin plots from the ggplot2 R package were generated.

## Results

### Lipidome During Early Life

**FIGURE 1:**
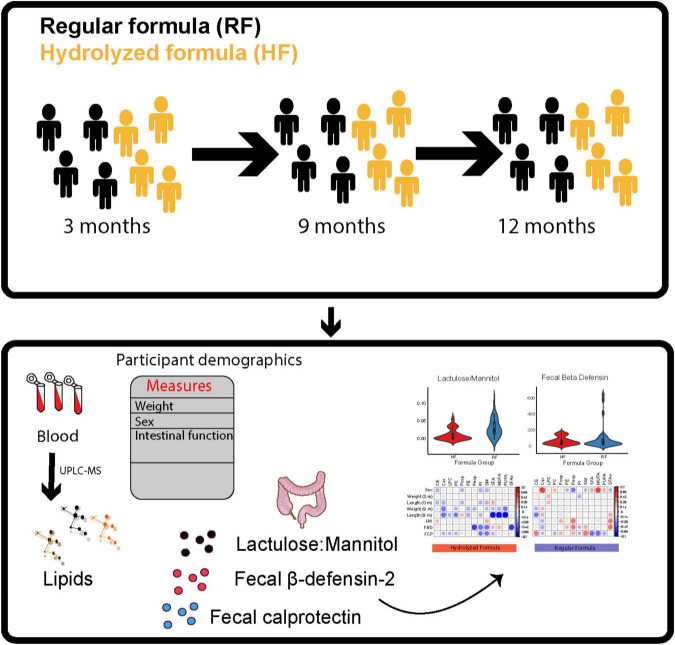
An overview of the study setting. Serum samples for lipidomic analysis were obtained from the Early Dietary Intervention and Later Signs of Beta-Cell Autoimmunity: Potential Mechanisms (EDIA) study which is a small-scale intervention trial comparing weaning infants onto an extensively hydrolyzed milk formula vs. a conventional cow’s milk-based formula. The study groups were matched by human leucocyte (HLA)-associated diabetes risk and period of birth. For each child, longitudinal samples were obtained corresponding to the ages of 3, 9, and 12 months. These age groups were selected with the objective of understanding the longitudinal lipid profiles in infants change after ingesting extensively hydrolyzed infant formula or conventional regular infant formula. Only, those children with information about amount of study formula per day were included in the data analysis.

We analyzed serum lipids from infants in two study groups: 40 who consumed conventional RF and 33 who consumed HF. Up to three longitudinal samples per child were collected, corresponding to the ages of 3, 9, and 12 months (Figure 1). The intervention study groups were matched based on HLA-associated diabetes risk. In total, 205 blood samples were analyzed for this study, and 181 lipid species were identified from nine major lipid classes including triacylglycerols (TGs), ceramides (Cer), cholesterol esters (CEs), diacylglycerols (DGs), lysophosphatidylcholines (LPCs), phosphatidylcholines (PCs), phosphatidylethanolamines (PEs), phosphatidylinositols (PIs), and sphingomyelins (SMs).

### Impact of Infant Milk Formula Type on the Circulating Metabolome and Microbiome

**FIGURE 2:**
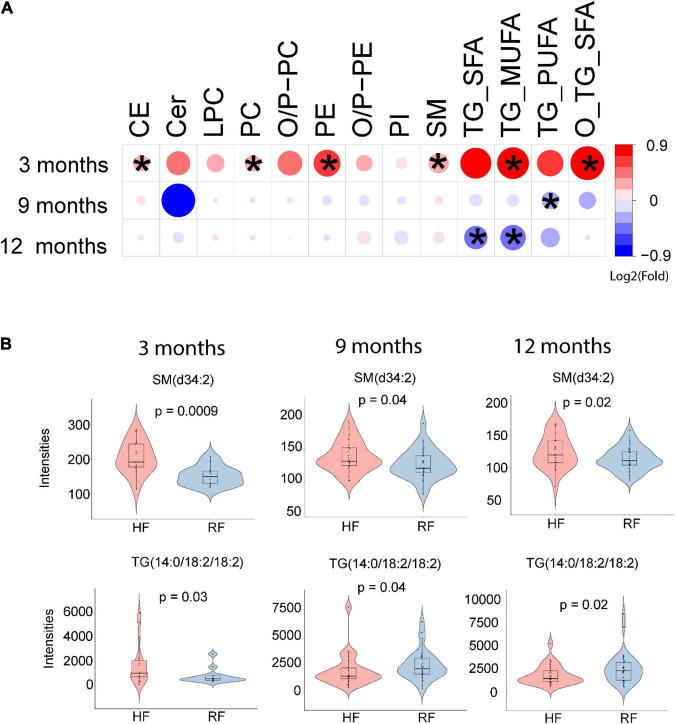
Comparison of lipidome between the infants who consumed an extensively hydrolyzed milk formula vs. a conventional cow’s milk-based formula. **(A)** Total lipid concentration differences in each lipid class between the intervention groups. **(B)** Box plot showing selected lipid species representative of lipid classes that change at 3, 9, and 12 months of age when comparing the study groups. Here, HF stands for extensively hydrolyzed infant formula and RF for conventional regular infant formula.

Infant milk formula had a marked impact on the infants’ circulating lipidome (Figure 2). SMs, PCs, and TGs were differentially impacted across the two formula intervention groups (Figure 2A). Serum concentrations of 68 molecular lipids were higher in the HF group as compared to the RF group at 3 months of age (*p* < 0.05, Figure 2 and Supplementary Figure 1), including two CEs, two LPCs, 32 PCs, three PEs, nine SMs, and 20 TGs (Supplementary Table 1).

Next, we analyzed lipid concentration differences between the two intervention groups in the remaining longitudinal series. Among the SMs, SM(d34:2) concentration remained higher in the HF group as compared to the RF group at 9 and 12 months of age (Figure 2B and Supplementary Figure 2). During the same period, TGs were observed to be lower in the HF group. Specifically, TGs of low acyl saturation (TG_SFA, with 0-1 double bonds/fatty acyl, i.e., saturated or monounsaturated fatty acids) and those with maximum two double bonds/fatty acyl (TG_MUFA) were altered at 12 months of age. TGs containing multiple double bonds (TG_PUFA) were altered at 9 months of age (Figure 2). At 9 months of age, we observed that a total of 19 TGs were different between the two study groups (Supplementary Figure 2 and Supplementary Table 2). In addition, we found that PC (36:2) and SM (d33:1) remained higher in the HF group at 9 months of age. At 12 months of age, two PCs and 46 TGs remained higher, whereas two SMs, one plasmalogen {PC [18:0p/18:1(9Z)]}, and another ether PC were lower in the RF group (Supplementary Figure 3 and Supplementary Table 3). Besides the intervention effect, linear models revealed that longitudinal lipid levels were affected by sex (male vs. female) and the amount of study formula intake (Supplementary Figures 1–3). Pairwise comparison between 3 and 9 months of age revealed that LPCs and SMs decreased in levels in the HF group with age, whereas CE, PE, (O/P)-PC, PI, TG_MUFA, TG_SFA, and TG_PUFA decreased in the RF group (Supplementary Figure 4).

We also analyzed the two milk formulas and found that 83 lipids, mainly SMs, LPCs, and PCs, differed between the HF and RF formulas (Supplementary Figure 5). In addition, we performed intention to treat analysis, which involves analysis among all randomized subjects regardless they were exposed to intervention formula or not (lipids ∼ sex + case). We found similar results to that of analysis when only those children with information about the AFPD were included (Supplementary Figure 6).

Similarly, we analyzed the circulating bile acid and polar metabolite concentrations in both intervention groups at 3 months of age. No differences were detected between the intervention groups, with the exception for arachidic acid, which was found to be lower in the RF compared to the HF group (Supplementary Figure 7). Next, we set out to determine whether the relative abundance of specific gut microbes changed in the intervention group. Levels of *Granulicatella*, *Enterococcaceae*, *Bifidobacteriales*, *Alloscardovia*, and *Propionimicrobium* were higher, whereas *Eggerthella* was lower in the RF as compared to the HF group at 3 months of age (Supplementary Figure 8). Taxon set enrichment analysis using Microbiome Analyst (PMID: 31942082) showed that these microbial changes were similar to those found in treatment with prebiotic consumption.

### Association Among Circulating Lipids, Infant Growth, and Intestinal Function

**FIGURE 3:**
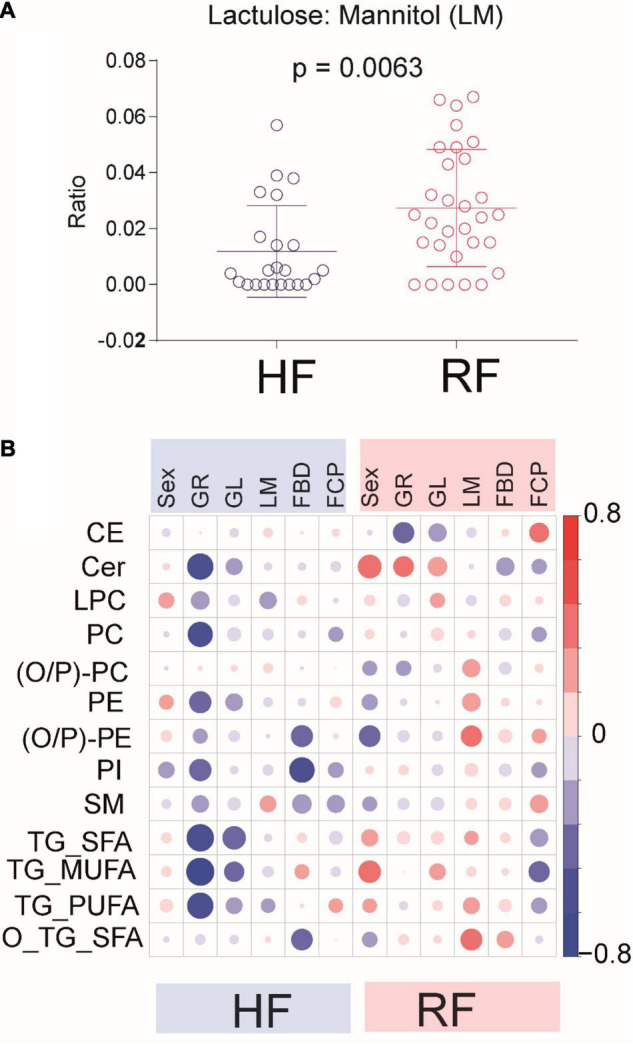
Associations between serum lipids and gastrointestinal markers in the infants. **(A)** Comparison of lactulose mannitol ratio between HF vs. RF infants at 9 months of age. **(B)** Pairwise Spearman correlations as calculated between serum lipids and offspring gut inflammation marker and permeability. Here, FBD stands for fecal beta-defensin and FCP for fecal calprotectin. Correlations were calculated between simultaneous measurements at 9 months of age. Positive correlations marked in red, inverse correlations marked in blue. Dot size for each pairwise correlation corresponds to the strength of the calculated correlation.

Hydrolyzed infant formula was found to associate with infants’ intestinal function (Figure 3). The comparison of lactulose mannitol (LM) ratio, fecal calprotectin (FCP), and fecal β-defensin (FBD) between the formula groups revealed higher LM ratio in children who consumed RF as compared to the HF group at 9 months of age (Figure 3A). However, no clear differences with respect to FCP or FBD were observed in any specific age group.

Given the known role of intestinal permeability in the intestinal maturation process and in the establishment of the immune system in infants, we also investigated the associations between circulating lipid levels, intestinal function, and infant growth at 9 months of age. To reduce dimensionality of the lipidomic data and to facilitate identification of global associations, we determined lipid class-specific associations between infant growth and intestinal function markers. Notably, the pattern of association between infant clinical factors including LM ratio and lipid class, as observed in the RF group, was absent in the HF group (Figure 3B). There was a clear, opposite association trend between some phospholipid classes [(O/P)-PC, (O/P)-PE], O-TG_SFA, and markers of intestinal function (LM).

Next, we performed correlation analysis between clinical variables and individual serum lipid levels. In the RF group, the analysis at the level of individual lipids revealed a total of nine lipid species, including PC(31:0), PC(O-40:5), PC(O-40:4), PC(O-38:4), PC(O-38:5), PC(36:2), PE(O-38:5) or PE(P-38:4), TG(O-52:2) or TG(P-52:1), and TG(54:2), were positively associated with the LM ratio at 9 months of age (Supplementary Table 4). However, no association between individual lipids and intestinal permeability was observed in the HF group. In the RF group, FCP and FDP were associated with several individual lipids (SMs, TGs, and PCs; Supplementary Table 4). This trend was less pronounced in the HF group. Besides permeability, Cer(d18:1/23:0) and Cer (d18:1/22:0) were positively associated with the child’s growth in the RF group. In the HF group, direct associations with growth were detected for Cer(d18:1/22:0) and multiple TGs (Supplementary Table 5).

## Discussion

Herein, we demonstrate that the longitudinal serum lipidome is different in infants fed HF as compared to infants fed RF. Specifically, SMs and TGs contributed to the differences between the intervention groups at 3, 9, and 12 months of age. SMs were persistently higher in the HF group, whereas TGs species were lower in HF at 9 and 12 months of age. PCs and LPCs also contributed to the separation between the intervention groups.

There is a general consensus that the infant metabolome is strongly correlated with feeding modality (18, 34). An earlier metabolomic study reported that plasma lipids, specifically PCs and SMs, are affected by infant feeding status during early life (34). Our results are consistent with these previous reports since we observed differences in serum concentrations of SMs between the two formulas (HF vs. RF). We observed higher level of SMs in the HF-fed group in our longitudinal setting. Sphingolipids are the main source of choline and are the major membrane lipid components of the mammalian cells (22, 35). SMs account for a major proportion (∼37%) of the phospholipid fraction in human’s breast milk and thus have a crucial role in infant nutrition (36, 37). Previous studies have shown that reduced levels of SMs in blood in early life are associated with the appearance of beta-cell autoimmunity and progression to type 1 diabetes later in life (21, 22). More recent results also indicate that treatment with fenofibrate raises the levels of sphingolipids and prevents autoimmune diabetes in mice (38). Furthermore, Sari et al. showed that a low carbohydrate diet increases blood SMs in patients with type 1 diabetes, which are thought to reduce dyslipidemia (38). We thus propose that dietary intervention-based targeted lipid alteration should be considered as a preventive strategy in type 1 diabetes. Clearly, larger clinical intervention studies are needed to test this hypothesis.

Lysophosphatidylcholines are the bioactive lipids derived from PC, which modulate various biological processes in the mammalian cells, including cytotoxic T-cell responses (39–41). Here, we found that the concentration of LPCs decreased between 3 and 9 months of age in the HF formula group. Given the immunomodulatory action of LPCs (41), this may be considered as a favorable outcome of the HF diet. Similarly, reduced level of TGs suggests low-energy demand in HF-fed infants as compared to the RF group (42). In particular, the HF-fed infants had low level of O-TG_SFA, which could be a positive feature in terms of a potential beneficial effect on cholesterol metabolism (43, 44).

The lipid composition of infant formulas may vary significantly, depending on formula type (45). The differences in the amount of lipid species between the infant formulas can also imply differences in serum lipid content in the infants. For example, SM-fortified infant formula increases plasma SM levels (37). Moreover, our results also showed that lipid content differed between the two infant formulas (HF vs. RF).

We further investigated whether serum lipid composition was associated with intestinal function in the infants, given prior evidence that gut barrier permeability, systemic inflammation, and lipid metabolism are the complexly interrelated variables in various health and disease conditions (46). Intriguingly, we observed that the serum lipidome (mainly ether-linked lipids) and intestinal permeability were inversely correlated in RF-fed infants, whereas no such association was observed in HF infants. Dysregulated gut morphology and also reduced microbial abundance were previously found as a consequence of formula feeding when compared to breast milk (47, 48). Changes in gut microbial composition also alter gut permeability, which leads to the leakage of pro-inflammatory molecules into circulation (49, 50). Thus, the difference in pattern we observed between HF vs. RF suggests that increased intestinal permeability may be dysregulating the circulatory lipids in infants. Accordingly, modifications to RF are desirable to improve the gut morphology, which may consequently facilitate the composition and diversity of the gut and promote immune system development (48–50). Interestingly, our observations show that gut microbiome composition differed between the infants fed HF as compared to RF-fed infants. Recent studies also indicate that hydrolyzed milk protein has a modest effect on the gut microbiome composition (51, 52). Taken together, it is plausible that different dietary protein sources in early life may influence gut microbiotal composition and circulating lipids; however, the underlying mechanisms remain unclear. We acknowledge the main limitation of our study as its small sample size, which was not amenable to elucidating the impact of modified milk protein intervention on the infant microbiome.

## Conclusion

The hypothesis that dietary interventions can prevent progression to islet autoimmunity and type 1 diabetes remains controversial (11, 15). Despite such controversy, our result suggests that it may be possible, by dietary intervention in early infancy, to avoid the occurrence of a profile of lipid species associated with the development of beta-cell autoimmunity. Further studies are, however, warranted. Taken together, our results demonstrate a profound impact of hydrolyzed formula on the infant lipidome as compared to regular formula. Thus, the use of a lipidomic approach in nutritional intervention studies could help unravel potentially novel biomarkers related to infant nutrition.

## Data Availability Statement

The metagenomics datasets presented in this study can be found in online repositories. The names of the repository/repositories and accession number(s) can be found below: SRA, PRJNA475246. The lipidomic datasets generated in this study will be submitted to the Metabolomics Workbench repository (https://www.metabolomicsworkbench.org).

## Ethics Statement

The studies involving human participants were reviewed and approved by the Ethics and Research Committee of the participating Universities and Hospitals approved the study protocol. The study was conducted according to the guidelines in the Declaration of Helsinki. Clinical trial registration number: Clinicaltrials.gov Identifier: NCT01735123. Written informed consent to participate in this study was provided by the participants’ legal guardian/next of kin.

## Author Contributions

MK and MO conceived and designed the experiments. TR, TH, HS, MS, SV, and JI performed the experiments and acquired the data required for the study. SL and TH analyzed the data. SL, MO, and TH wrote the manuscript. All authors reviewed and revised the manuscript and approved the submitted version.

## Conflict of Interest

The authors declare that the research was conducted in the absence of any commercial or financial relationships that could be construed as a potential conflict of interest.

## Publisher’s Note

All claims expressed in this article are solely those of the authors and do not necessarily represent those of their affiliated organizations, or those of the publisher, the editors and the reviewers. Any product that may be evaluated in this article, or claim that may be made by its manufacturer, is not guaranteed or endorsed by the publisher.

## Footnotes

1
https://www.r-project.org/
