# Supplementary material for: Impact of Extensively Hydrolyzed Infant Formula on Circulating Lipids During Early Life
Source: Front Nutr. 2022 May 24;9:859627. doi: 10.3389/fnut.2022.859627 (PMC9171511; doi:10.3389/fnut.2022.859627)
Supplement: Supplementary file 2 [file Data_Sheet_1.PDF]

Supplementary Figure

Figure S1

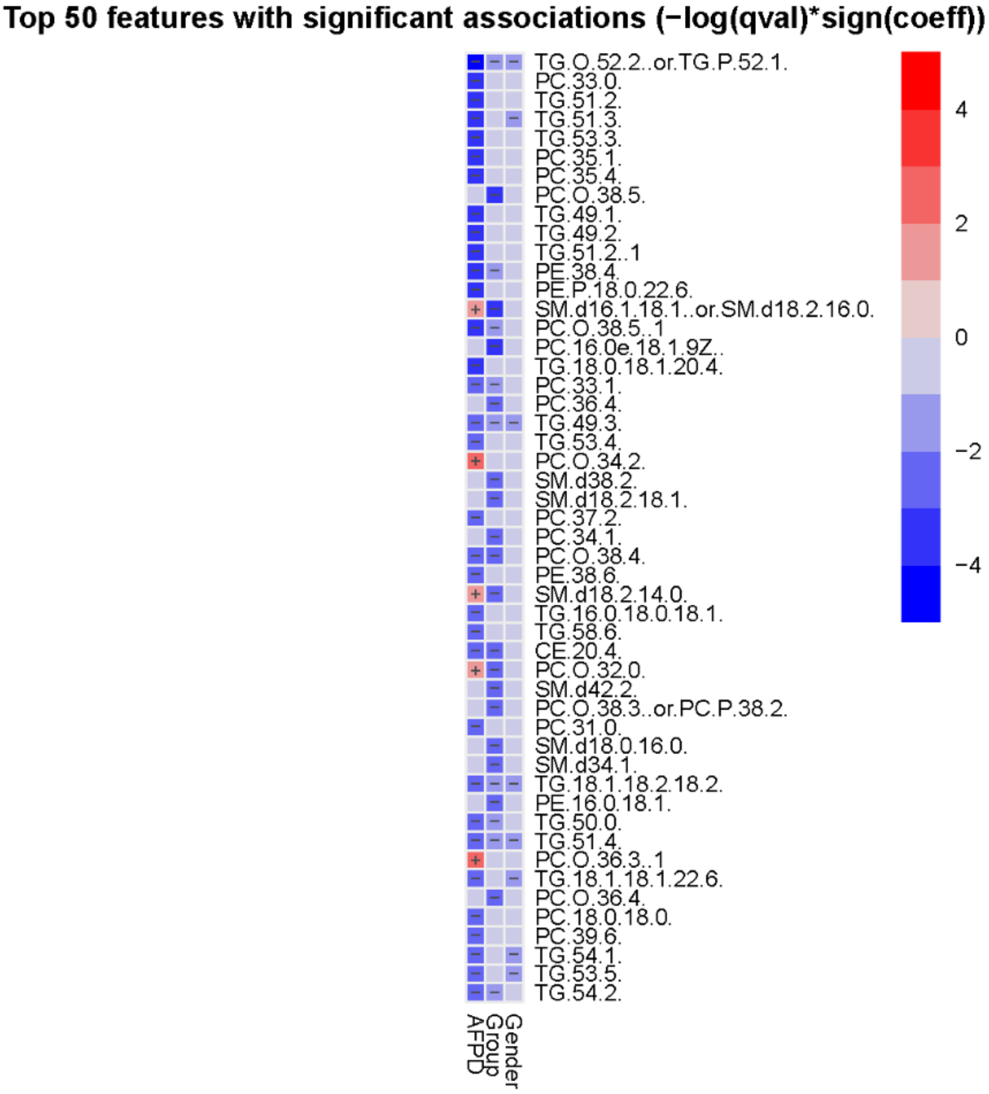

Figure S2

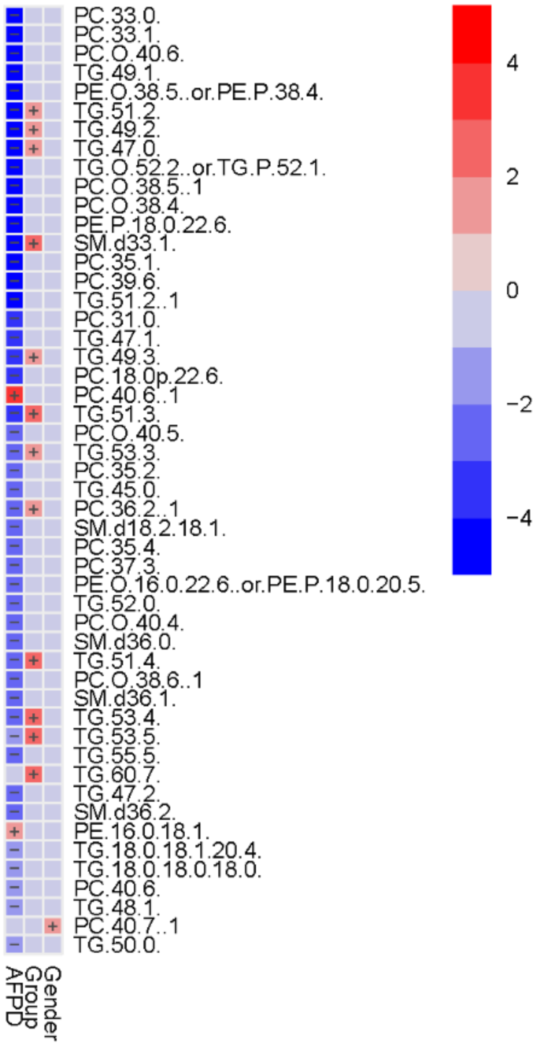
Top 50 features with significant associations (−log(qval)*sign(coeff))
PC.33.0.
PC.33.1.
PC.O.40.6.
TG.49.1.
PE.O.38.5..or.PE.P.38.4.
TG.51.2.
TG.49.2.
TG.47.0.
TG.O.52.2..or.TG.P.52.1.
PC.O.38.5..1
PC.O.38.4.
PE.P.18.0.22.6.
SM.d33.1.
PC.35.1.
PC.39.6.
TG.51.2..1
PC.31.0.
TG.47.1.
TG.49.3.
PC.18.0p.22.6.
PC.40.6..1
TG.51.3.
PC.O.40.5.
TG.53.3.
PC.35.2.
TG.45.0.
PC.36.2..1
SM.d18.2.18.1.
PC.35.4.
PC.37.3.
PE.O.16.0.22.6..or.PE.P.18.0.20.5.
TG.52.0.
PC.O.40.4.
SM.d36.0.
TG.51.4.
PC.O.38.6..1
SM.d36.1.
TG.53.4.
TG.53.5.
TG.55.5.
TG.60.7.
TG.47.2.
SM.d36.2.
PE.16.0.18.1.
TG.18.0.18.1.20.4.
TG.18.0.18.0.18.0.
PC.40.6.
TG.48.1.
PC.40.7..1
TG.50.0.
AFPD
Group
Gender
4
2
0
−2
−4

Figure S3

Top 50 features with significant associations (−log(qval)*sign(coeff))

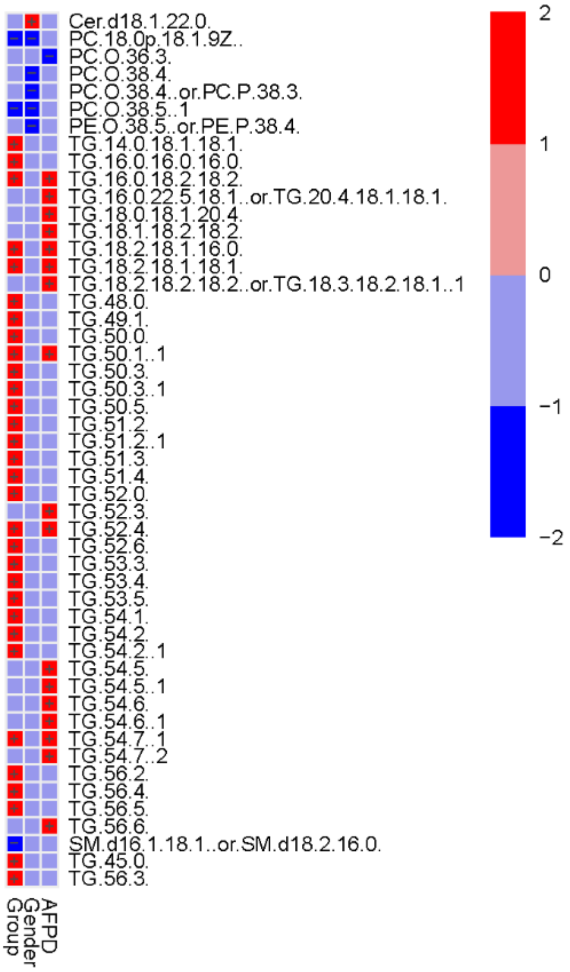

Figure S4

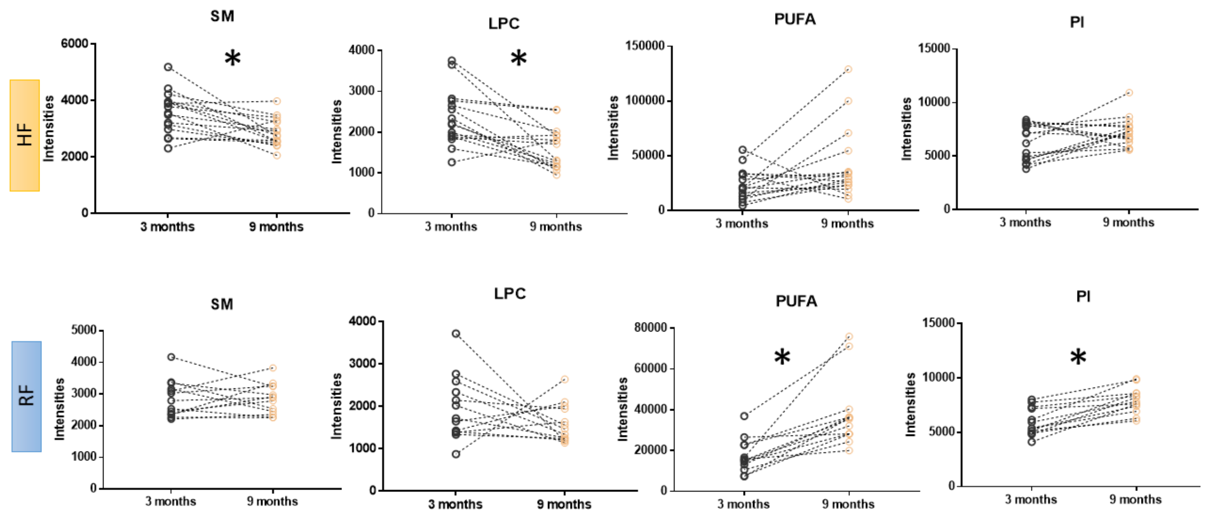

Figure S5

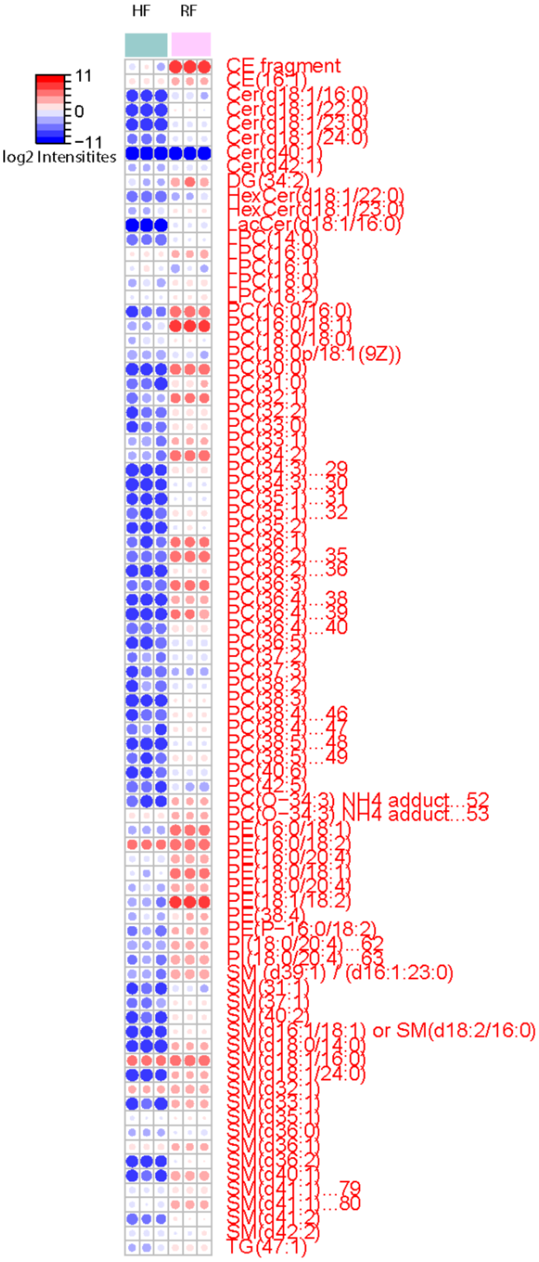
HF
RF
11
0
−11
log2 Intensitites
CE fragment
CE(16:1)
Cer(d18:1/16:0)
Cer(d18:1/22:0)
Cer(d18:1/23:0)
Cer(d18:1/24:0)
Cer(d40:1)
Cer(d42:1)
DG(34:2)
HexCer(d18:1/22:0)
HexCer(d18:1/23:0)
LacCer(d18:1/16:0)
LPC(14:0)
LPC(16:0)
LPC(16:1)
LPC(18:0)
LPC(18:2)
PC(16:0/16:0)
PC(16:0/18:1)
PC(18:0/18:0)
PC(18:0p/18:1(9Z))
PC(30:0)
PC(31:0)
PC(32:1)
PC(32:2)
PC(33:0)
PC(33:1)
PC(34:2)
PC(34:3)...29
PC(34:3)...30
PC(35:1)...31
PC(35:1)...32
PC(35:2)
PC(36:1)
PC(36:2)...35
PC(36:2)...36
PC(36:3)
PC(36:4)...38
PC(36:4)...39
PC(36:4)...40
PC(36:5)
PC(37:2)
PC(37:3)
PC(38:2)
PC(38:3)
PC(38:4)...46
PC(38:4)...47
PC(38:5)...48
PC(38:5)...49
PC(40:6)
PC(42:5)
PC(O−34:3) NH4 adduct...52
PC(O−34:3) NH4 adduct...53
PE(16:0/18:1)
PE(16:0/18:2)
PE(16:0/20:4)
PE(18:0/18:1)
PE(18:0/20:4)
PE(18:1/18:2)
PE(38:4)
PE(P−16:0/18:2)
PI(18:0/20:4)...62
PI(18:0/20:4)...63
SM (d39:1) / (d16:1:23:0)
SM(31:1)
SM(37:1)
SM(40:2)
SM(d16:1/18:1) or SM(d18:2/16:0)
SM(d18:0/14:0)
SM(d18:1/16:0)
SM(d18:1/24:0)
SM(d32:1)
SM(d33:1)
SM(d35:1)
SM(d36:0)
SM(d36:1)
SM(d36:2)
SM(d40:1)
SM(d41:1)...79
SM(d41:1)...80
SM(d41:2)
SM(d42:2)
TG(47:1)

Figure S6

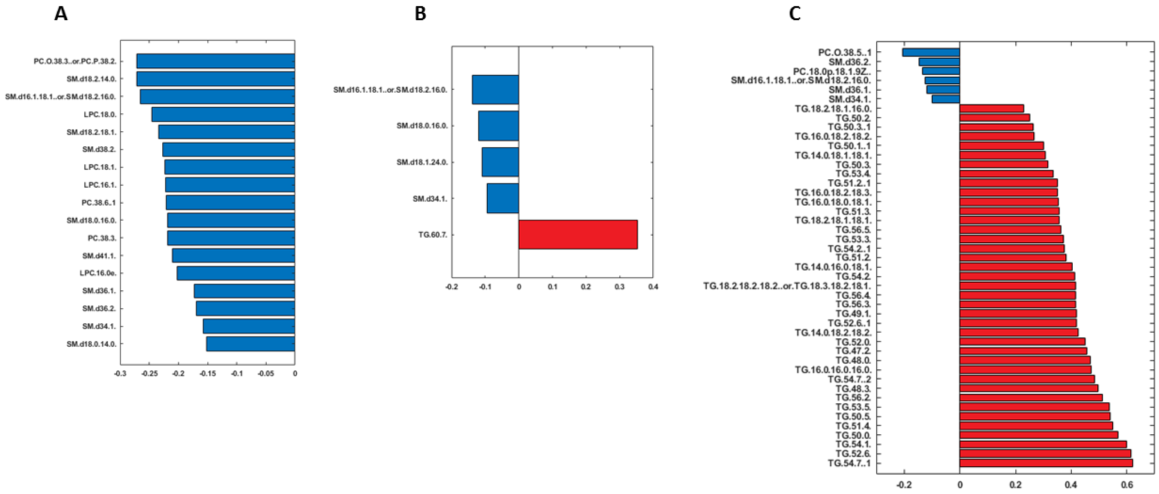
A
PC.O.38.3..or.PC.P.38.2.
SM.d18.2.14.0.
SM.d16.1.18.1..or.SM.d18.2.16.0.
LPC.18.0.
SM.d18.2.18.1.
SM.d38.2.
LPC.18.1.
LPC.16.1.
PC.38.6..1
SM.d18.0.16.0.
PC.38.3.
SM.d41.1.
LPC.16.0e.
SM.d36.1.
SM.d36.2.
SM.d34.1.
SM.d18.0.14.0.
-0.3 -0.25 -0.2 -0.15 -0.1 -0.05 0
B
SM.d16.1.18.1..or.SM.d18.2.16.0.
SM.d18.0.16.0.
SM.d18.1.24.0.
SM.d34.1.
TG.60.7.
-0.2 -0.1 0 0.1 0.2 0.3 0.4
C
PC.O.38.5..1
SM.d36.2.
PC.18.0p.18.1.9Z..
SM.d16.1.18.1..or.SM.d18.2.16.0.
SM.d36.1.
SM.d34.1.
TG.18.2.18.1.16.0.
TG.50.2.
TG.50.3..1
TG.16.0.18.2.18.2.
TG.50.1..1
TG.14.0.18.1.18.1.
TG.50.3.
TG.53.4.
TG.51.2..1
TG.16.0.18.2.18.3.
TG.16.0.18.0.18.1.
TG.51.3.
TG.18.2.18.1.18.1.
TG.56.5.
TG.53.3.
TG.54.2..1
TG.51.2.
TG.14.0.16.0.18.1.
TG.54.2.
TG.18.2.18.2.18.2..or.TG.18.3.18.2.18.1.
TG.56.4.
TG.56.3.
TG.49.1.
TG.52.6..1
TG.14.0.18.2.18.2.
TG.52.0.
TG.47.2.
TG.48.0.
TG.16.0.16.0.16.0.
TG.54.7..2
TG.48.3.
TG.56.2.
TG.53.5.
TG.50.5.
TG.51.4.
TG.50.0.
TG.54.1.
TG.52.6.
TG.54.7..1
-0.2 0 0.2 0.4 0.6

Figure S7

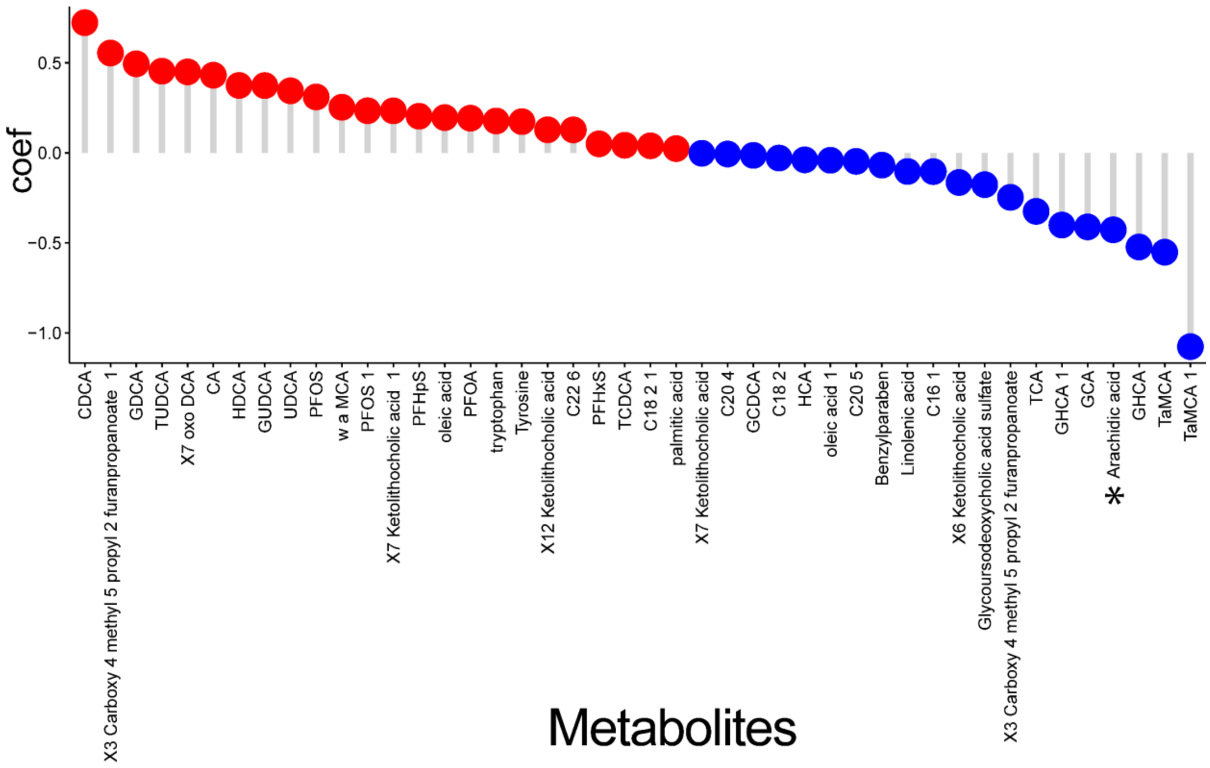
coef
0.5
0.0
−0.5
−1.0
CDCA
X3 Carboxy 4 methyl 5 propyl 2 furanpropanoate 1
GDCA
TUDCA
X7 oxo DCA
CA
HDCA
GUDCA
UDCA
PFOS
w a MCA
PFOS 1
X7 Ketolithocholic acid 1
PFHpS
oleic acid
PFOA
tryptophan
Tyrosine
X12 Ketolithocholic acid
C22 6
PFHxS
TCDCA
C18 2 1
palmitic acid
X7 Ketolithocholic acid
C20 4
GCDCA
C18 2
HCA
oleic acid 1
C20 5
Benzylparaben
Linolenic acid
C16 1
X6 Ketolithocholic acid
Glycoursodeoxycholic acid sulfate
X3 Carboxy 4 methyl 5 propyl 2 furanpropanoate
TCA
GHCA 1
GCA
* Arachidic acid
GHCA
TaMCA
TaMCA 1
Metabolites

Figure S8

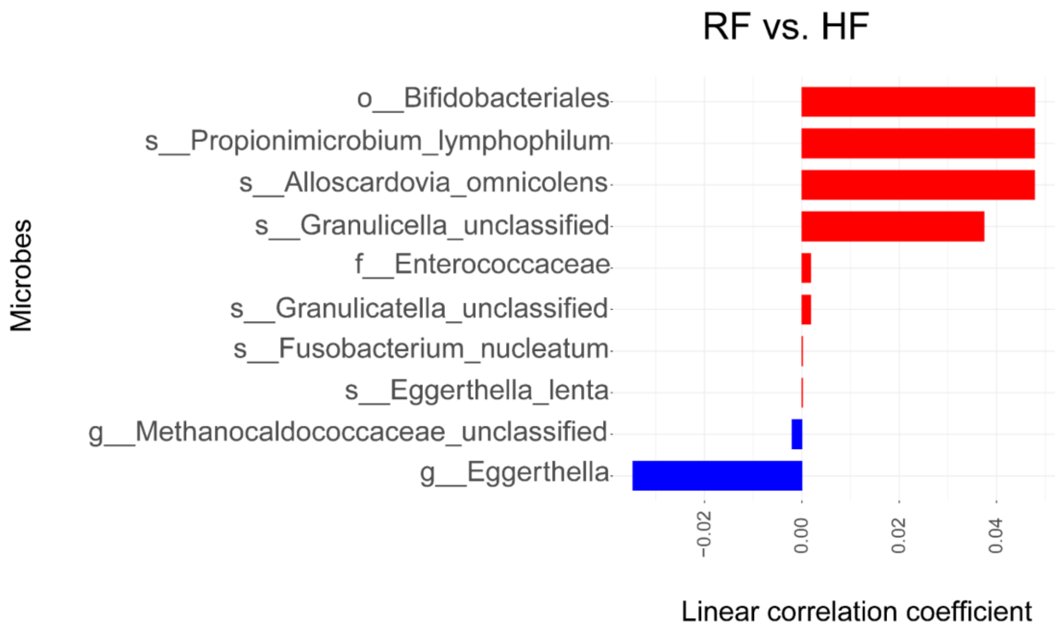

RF vs. HF
Microbes
o__Bifidobacteriales
s__Propionimicrobium_lymphophilum
s__Alloscardovia_omnicolens
s__Granulicella_unclassified
f__Enterococcaceae
s__Granulicatella_unclassified
s__Fusobacterium_nucleatum
s__Eggerthella_lenta
g__Methanocaldococcaceae_unclassified
g__Eggerthella
−0.02
0.00
0.02
0.04
Linear correlation coefficient

Supplementary Figure captions

Figure S1-S3. Comparison of lipidome between the infants who consumed an extensively hydrolyzed milk formula vs. a conventional cow's milk-based formula at 3 months (Figure S1), 9 months (Figure S2) and 12 months (Figure S3) of age respectively. The difference in the lipidome between two intervention group were compared using multivariable linear model with the fixed effect being case (Hydrolyzed formula vs Regular Formula), co-accounting for sex (male vs. female) and amount of study formula per day (AFPD) using MaAsLin2 package in R. Only those children with information about the amount of study formula per day were included in the multivariable linear comparative analysis. The plot shows the most discriminating lipids between the two intervention groups (RF vs. HF) compared using multivariable linear model.

Figure S4. Total lipid concentration in each lipid class difference between two different intervention timeframe. The pairwise plot showing the most discriminating lipids clusters between the samples obtained at 3 months and 9 months of age from same individual. The result from paired t-test shows that LPCs and SMs decreased in the HF group with age, while CE, PE, (O/P)-PC, PI, TG_MUFA, TG_SFA, and TG_PUFA decreased in the the RF group.

Figure S5. The most discriminating lipids between the two milk formulas. We found that 83 lipids, mainly SMs, LPCs and PCs, differed between the HF and RF formulas.

Figure S6. Comparison of lipidome between the infants who consumed an extensively hydrolyzed milk formula vs. a conventional cow's milk-based formula at 3 months (A), 9 months (B) and 12 months (C) of age respectively. Here, we performed included all randomized subjects regardless they were exposed to intervention formula or not (lipids ~ sex +case). This intention to treat analysis is a strategy for analyzing results in a prospective randomized study. The plot shows the most discriminating lipids between the two intervention groups (RF vs. HF) compared using multivariable linear model.

Figure S7. The difference in the polar metabolites between two intervention groups (HF vs RF) compared using multivariable linear model.

Figure S8. The difference in the microbiome abundances between two intervention groups (Hydrolyzed formula vs Regular Formula) compared using multivariable linear model at 3 months of age.

Supplementary Table captions

Supplementary Table 1-3. Information about the molecular lipids that were different between the HF group as compared to the RF group at 3 months (Supplementary Table 1), 9 months (Supplementary Table 2) and 12 months (Supplementary Table 3) of age respectively.

Supplementary Table 4-5. Correlation analysis between clinical variables and individual serum lipids level in the Regular Formula group (Supplementary Table 4) and Hydrolyzed Formula (Supplementary Table 5) respectively. The association between the lipidome and clinical variable was computed using multivariable linear model between two intervention groups.
